# Oral Health—A Neglected Aspect of Subjective Well-Being in Later Life

**DOI:** 10.1093/geronb/gbw024

**Published:** 2016-03-12

**Authors:** Patrick Rouxel, Georgios Tsakos, Tarani Chandola, Richard G Watt

**Affiliations:** 1 UCL Eastman Dental Institute, London, UK; 2 Department of Epidemiology and Public Health, University College London, London, UK; 3 Cathie March Institute for Social Research, University of Manchester, Manchester, UK

**Keywords:** Aging, Depressive symptoms, Edentulous, Oral health, Quality of life, Subjective well-being

## Abstract

**Objectives:**

This study examined whether oral health is a neglected aspect of subjective well-being (SWB) among older adults. The key research question was whether deterioration in oral health among dentate older adults living in England was associated with decreases in SWB, using measures of eudemonic, evaluative, and affective dimensions of well-being.

**Methods:**

This secondary analysis used data from the third (2006–2007) and fifth (2010–2011) waves of respondents aged 50 and older from the English Longitudinal Study of Ageing (ELSA). We fitted multivariable regression models to examine the effects of changes in oral impacts on daily life and edentulism (complete tooth loss) on SWB (quality of life, life satisfaction, and depressive symptomatology).

**Results:**

A worsening in both oral health measures was associated with an increase in depressive symptoms even after adjusting for time-varying confounders including declining health, activities of daily living, and reduced social support. Becoming edentate was also associated with decreases in quality of life and life satisfaction.

**Discussion:**

A deterioration in oral health and oral health–related quality of life increases the risk of depressive symptoms among older adults and highlights the importance of oral health as a determinant of subjective well-being in later life.

The global burden of oral conditions increased from 1990 to 2010 mainly due to population growth and aging, with untreated caries being the most prevalent of all diseases and injuries (Marcenes et al., 2013). In Britain, substantial proportions of older adults experience problems with their daily life due to the condition of their mouth and dentition (White et al., 2012).

Despite the high prevalence of oral conditions among older adults, oral health is seldom explicitly examined in epidemiological studies of well-being and aging (Cole & Dendukuri, 2003; Vink, Aartsen, & Schoevers, 2008). In a review of risk factors for depression among the elderly, oral health was not mentioned in any of the 80 studies reviewed (Vink et al., 2008). Yet oral problems, particularly tooth loss and edentulism, have been linked to the risk of depressive symptoms (O’Neil et al., 2014; Takiguchi, Yoshihara, Takano, & Miyazaki, 2015) and different domains of quality of life and well-being (Fontanive, Abegg, Tsakos, & Oliveira, 2013; Hassel et al., 2011; Hugo, Hilgert, de Sousa Mda, & Cury, 2009; Rodrigues, Oliveira, Vargas, Moreira, & Ferreira, 2012). However, these studies have methodological limitations, such as their cross-sectional design, small sample sizes, and inadequate control for key potential confounders like disability, which increase with age.

The research question of this study was: Is a worsening in oral health among older adults associated with adverse changes in their subjective well-being (SWB), even after accounting for other factors associated with lower levels of well-being in later life?

## Method

### Data

This was a secondary analysis of data from the third (2006–2007) and fifth (2010–2011) waves of the English Longitudinal Study of Ageing (ELSA). ELSA is a panel study of people aged 50 and older, which began in 2002, with a follow-up every 2 years. Baseline and follow-up data on oral health came from Waves 3 and 5, when oral health was assessed in ELSA.

There were 8,552 noninstitutionalized respondents who completed interviews at Wave 3 and 6,793 respondents at Wave 5. Only respondents who completed the interviews in person at both these waves were eligible for the analysis (*N* = 6,294).

### Dependent Variables: Subjective Well-Being

*Evaluative well-being* was measured using the 5-item Satisfaction With Life scale (Diener, Emmons, Larsen, & Griffin, 1985). Responses were summed to provide a score from 5 to 35, with higher scores indicating greater life satisfaction. The measure possesses a good degree of internal consistency (MacKinnon & Keating, 1989).

*Affective well-being* was not measured directly. Instead, a shortened 8-item version of the Center for Epidemiologic Studies–Depression (CES-D) scale (Radloff, 1977) provided a measure of negative affect during the last week due to depressive symptoms. The items were summed to give the extent of depressive symptoms ranging from 0 to 8. The 8-item version has good internal consistency and other psychometric properties comparable with the full 20-item CES-D scale (Steffick, 2000). The CES-D scores were also dichotomized at the point of three or more symptoms to indicate those most at risk of depression (Blane, Netuveli, & Montgomery, 2008).

*Eudemonic well-being* was measured using the CASP-19 scale. The CASP-19 measures quality of life in later age related to four domains: control (C), autonomy (A), self-realization (S), and pleasure (P) with validated psychometric properties (Hyde, Wiggins, Higgs, & Blane, 2003). Likert-type responses to each item (scoring 0–3) were summed to provide an overall quality-of-life score, ranging from 0 to 57, where higher scores indicate better quality of life.

### Independent Variables

*Edentulism* was measured by self-reports of the presence/absence of natural teeth and grouping respondents into dentate (with natural teeth) versus edentate (without any).

*Oral health–related quality of life* (OHRQoL) was measured through a modified version of the Oral Impacts on Daily Performances (OIDP) questionnaire, a valid and reliable measure of OHRQoL among older adults (Tsakos, Marcenes, & Sheiham, 2001). Difficulties in eating, speaking, smiling, and problems with emotional stability or with socializing due to their teeth, mouth, or dentures (in the past 6 months) were grouped into reports of at least one oral impact against none.

Control variables were selected based on a previous study identifying the key predictors of subjective well-being (Jivraj et al., 2014). In our study, variables that were associated (*p* < .05) with oral health indicators and SWB included age, gender, cohabiting status, economic activity (unemployed, permanently sick or disabled, or looking after family), quintiles of nonpension household wealth, self-rated health, smoking, social support (perceived emotional support from spouse/partner, children, other relatives, and friends), and disability (activities of daily living and instrumental activities of daily living, ADL/IADL).

### Data Analysis

We created measures of change by subtracting the Wave 5 score from the Wave 3 score for continuous variables. For categorical variables (OIDP and CES-D using the cutoff of three), the change between two measurements was reduced to three categories for the analysis: no change, incident oral impacts/depressive symptoms, and recovery from oral impacts/depressive symptoms. For edentulism, the only possible change between the waves was to remain dentate or become edentate. The edentate at baseline was dropped from further analysis.

For descriptive analyses, we compared the mean SWB change scores by changes in the time-varying covariates (cohabiting status, economic activity, general health, ADL/IADL, and social support) and in the time-invariant covariates (age at Wave 3, gender, smoking, and wealth). There was little change in smoking and wealth quintiles between waves so the Wave 3 values were used.

We regressed the change in well-being continuous variables on each of the oral health variables separately. We used multinomial logit regression models to examine the association of changes in oral health with incident depressive symptoms or recovery from depressive symptoms using the CES-D categories. Analyses reported in main manuscript are either bivariate associations between oral health and well-being (Model 1) or fully adjusted for covariates (Model 2).

## Results

The mean changes in SWB by change in the oral health measures and the covariates are displayed in Supplementary Table 1. Quality of life decreased by 0.61 CASP-19 points over 4 years, whereas life satisfaction increased by 0.75 points on the 30-point life satisfaction scale and depressive symptoms by just 0.06 on the 8-point CES-D scale.

Respondents with an incident oral impact reported a decrease in CASP-19 scores (−1.33; *p =* .065) and an increase in depressive symptoms (0.28; *p* < .001). Respondents who no longer had an oral impact also reported an improvement in their life satisfaction (1.13; *p* = .091) and a decrease in depressive symptoms (−0.22; *p* < .001). Becoming edentate was associated with negative changes in all three SWB measures.

Table 1 displays multiple regression models results with changes in the SWB measures as the dependent variables and changes in oral impacts and dentate status as the explanatory variables. In the bivariate models (Model 1), respondents with an incident oral impact were significantly more likely to report worsening in both their quality of life and depressive symptoms; there was no significant association between oral impact and reported life satisfaction. In Model 2, which adjusted for time-constant and time-varying covariates, the incidence of at least one oral impact was no longer significantly associated with a worsening in quality of life scores but was still associated with a significant increase of 0.26 (*p* < .01) in the CES-D scale. Respondents who no longer had an oral impact did not report a significant improvement in their SWB, although the regression coefficients were in the expected direction for life satisfaction (positive coefficients) and depressive symptoms (negative coefficients).

**Table 1.** Multiple Regression Models of Change in Subjective Well-Being Scores by Change in Oral Health Indicators Between Waves 3 (2006–2007) and 5 (2010–2011), Regression Coefficient (95% CI)

	Change in CASP-19 (*N* = 4,472)			Change in life satisfaction (*N* = 4,608)			Change in depressive symptoms (*N* = 4,807)		
	Model 1 (Coef [95% CI])	Model 2 (Coef [95% CI])	*n*	Model 1 Coef (95% CI (Coef [95% CI]))	Model 2 (Coef [95% CI])	*n*	Model 1 (Coef [95% CI])	Model 2 (Coef [95% CI])	*n*
Change in OIDP									
No change	0.00	0.00	3,966	0.00	0.00	4,084	0.00	0.00	4,261
No impact to impact	−0.79* (−1.50, −0.09)	−0.64 (−1.31, 0.04)	310	−0.58 (−1.18, 0.02)	−0.52 (−1.10, 0.06)	314	0.29** (0.11, 0.47)	0.26** (0.09, 0.43)	327
Impact to no impact	−0.25 (−1.13, 0.62)	−0.09 (−0.92, 0.75)	196	0.61 (−0.11,1.34)	0.60 (−0.1, 1.3)	210	−0.16 (−0.37, 0.06)	−0.12 (−0.34, 0.09)	219
*R* ^2^	0.001	0.101		0.001	0.074		0.003	0.055	
	Change in CASP-19 (*N* = 3,966)			Change in life satisfaction (*N* = 4,061)			Change in depressive symptoms (*N* = 4,217)		
	Model 1 (Coef [95% CI])	Model 2 (Coef [95% CI])		Model 1 (Coef [95% CI])	Model 2 (Coef [95% CI])		Model 1 (Coef [95% CI])	Model 2 (Coef [95% CI])	
Change in edentulism									
No change	0.00	0.00	3,880	0.00	0.00	3,970	0.00	0.00	4,116
Becoming edentate	−2.13** (−3.44, −0.82)	−1.67** (−2.93, −0.42)	86	−2.54*** (−3.62, −1.46)	−2.20*** (−3.25, −1.15)	91	0.39* (0.08, 0.7)	0.33* (0.02, 0.63)	101
*R* ^**2**^	0.003	0.100		0.005	0.075		0.001	0.052	

*Notes*. ADL = activities of daily living; CASP-19 = quality of life; CI = confidence interval; IADL = instrumental activities of daily living; OIDP = Oral Impacts on Daily Performances. The reference category for change in CASP-19, Life Satisfaction and Depressive Symptoms is the “no change category.” Model 1: Bivariate model only including change in oral health. Model 2: Model 1 adjusted for sociodemographic (age-groups, gender, and cohabiting status), socioeconomic (economic activity status and wealth quintiles), health (self-rated health, ADL/IADL), and smoking and psychosocial (social support) factors.

*p < .05. **p < .01. ***p < .001.

Respondents who became edentate also reported worsening in all three SWB measures (Table 1), and these associations remained significant after adjusting for time-constant and time-varying covariates (Model 2). The detailed regression models showed that ADL/IADL confounded some of the associations between changes in oral health and changes in SWB (see Supplementary Tables 2–7).

Supplementary Table 8 reports the results of the logistic regression models with change in depressive symptoms using the cutoff of three on the CES-D scale. Older adults with an incident oral impact were more likely to become depressed, even after adjusting for time-varying and time-constant covariates (odds ratio [OR] = 1.60, 95% confidence interval [CI] = 1.20–2.15). Older adults who no longer had an oral impact had a reduced risk of incident depressive symptoms (adjusted OR = 0.62, 95% CI = 0.39–0.99). In contrast, recovery from depressive symptoms was not associated with changes in oral impact. Becoming edentate was associated with an increased risk of incident depressive symptoms, but this was no longer significant after adjusting for covariates (Model 2). Becoming edentate was associated with lower odds of recovering from depressive symptoms, but the association was not statistically significant.

## Discussion

The results show that a worsening in oral health was associated with a significant increase in depressive symptoms among older adults in England over a 4-year period. Moreover, becoming edentate was associated with significant decreases in quality of life and life satisfaction, whereas the incidence of oral impacts was no longer significantly associated with decreases in these two measures of well-being, once confounders were taken into account.

Changes in ADL/IADL were strongly related to changes in SWB as well as to changes in oral impacts. This is unsurprising, given that one of the ADL questions explicitly asks about limitations with eating (such as cutting up food). Although the focus of this ADL question is not supposed to be on problems related to chewing and swallowing (Katz, Ford, Moskowitz, Jackson, & Jaffe, 1963), responses to the ADL question on eating could overlap with the responses to the OIDP question on difficulty eating. Together with findings from other studies (Hassel et al., 2011; O’Neil et al., 2014), there is evidence that oral health is an important risk factor for well-being and depression among older adults.

Potential explanations for our findings include pathways leading from edentulism and poor OHRQoL to discomfort, pain, and functional limitations, which in turn might lead to physical and mood disorders and restriction in social activities (Fiske, Davis, Frances, & Gelbier, 1998; Steele et al., 2004). Futhermore, poor oral health is a risk factor for the progression of inflammatory diseases (Berk et al., 2013; de Oliveira, Watt, & Hamer, 2010), and depression has been linked to inflammatory disorders (Berk et al., 2013).

The longitudinal analysis presented in this study cannot conclusively establish the direction of the association between oral health and SWB. However, if poor oral health leads to depression, then the incidence of poor oral health should be associated with incident depression and a recovery from poor oral health should decrease the risk of depression. This is borne out by the results in Supplementary Table 2. If depression leads to poor oral health, then recovery from depression should be associated with a decreased risk of poor oral health. However, there was little evidence of such an association in Supplementary Table 2, as the odds of incident oral impacts increased when older adults recovered from depressive symptoms. These results suggest that the direction of the association from oral health to low SWB among older adults is more plausible than the reverse direction.

The prospective design of the ELSA study allowed us to examine changes in oral health and changes in SWB over a period of 4 years. However, attrition and nonresponse reduced the size of the analytical sample. A comparison with ELSA members without complete follow-up data showed that nonparticipants had lower well-being and poorer oral health than the participants. Previous analyses of oral health in ELSA (with a similar analytical sample) used multiple imputation for missing data, which led to results that were similar to those based on the complete sample (Rouxel et al., 2015).

There could also be other factors related to both well-being and oral health that were not included in the current study. For example, the findings could be partially explained by changes in life circumstances or personality traits that were not modeled in current analyses.

## Conclusion

Our study demonstrated that deterioration in oral health and oral health–related quality of life had a negative effect on depressive symptoms among older adults, suggesting the importance of oral health as a key determinant of SWB among older adults. Strategies to improve oral health among older adults may not only have direct benefits on their oral health but also have the potential to improve their well-being.

## Supplementary Material

Supplementary material can be found at: http://psychsocgerontology.oxfordjournals.org/

## Funding

Tarani Chandola was supported by the UK Economic and Social Research Council (grant number ES/J019119/1).

## Supplementary Material

Supplementary Tables 1-8

## References

[CIT0001] Berk M. Williams L. J. Jacka F. N. O’Neil A. Pasco J. A. Moylan S., … Maes M (2013). So depression is an inflammatory disease, but where does the inflammation come from?BioMed Central Medicine,11, 200. doi:10.1186/1741-7015-11-200 10.1186/1741-7015-11-200 PMC3846682 24228900

[CIT0002] Blane D. Netuveli G., & Montgomery S. M (2008). Quality of life, health and physiological status and change at older ages.Social Science & Medicine,66, 1579–1587. doi:10.1016/j.socscimed.2007.12.021 18241966 10.1016/j.socscimed.2007.12.021

[CIT0003] Cole M. G., & Dendukuri N (2003). Risk factors for depression among elderly community subjects: A systematic review and meta-analysis.The American Journal of Psychiatry,160, 1147–1156. doi:10.1176/appi.ajp.160.6.1147 12777274 10.1176/appi.ajp.160.6.1147

[CIT0004] de Oliveira C. M. Watt R. G., & Hamer M (2010). Toothbrushing, inflammation, and risk of cardiovascular disease: Results from Scottish Health Survey.British Medical Journal,340, 1–6. doi:10.1136/bmj.c2451 10.1136/bmj.c2451 PMC2877809 20508025

[CIT0005] Diener E. Emmons R. A. Larsen R. J., & Griffin S (1985). The Satisfaction With Life Scale.Journal of Personality Assessment,49, 71–75. doi:10.1207/s15327752jpa4901_13 16367493 10.1207/s15327752jpa4901_13

[CIT0006] Fiske J. Davis D. Frances C., & Gelbier S (1998). The emotional effects of tooth loss in edentulous people.British Dental Journal,184, 90–93. doi:10.1038/sj.bdj.4809551 9489217 10.1038/sj.bdj.4809551

[CIT0007] Fontanive V. Abegg C. Tsakos G., & Oliveira M (2013). The association between clinical oral health and general quality of life: A population-based study of individuals aged 50–74 in Southern Brazil.Community Dentistry and Oral Epidemiology,41, 154–162. doi:10.1111/j.1600-0528.2012.00742.x 22900534 10.1111/j.1600-0528.2012.00742.x

[CIT0008] Hassel A. J. Danner D. Schmitt M. Nitschke I. Rammelsberg P., & Wahl H. W (2011). Oral health-related quality of life is linked with subjective well-being and depression in early old age.Clinical Oral Investigations,15, 691–697. doi:10.1007/s00784-010-0437-3 20582443 10.1007/s00784-010-0437-3

[CIT0009] Hugo F. N. Hilgert J. B. de Sousa Mda L., & Cury J. A (2009). Oral status and its association with general quality of life in older independent-living south-Brazilians.Community Dentistry and Oral Epidemiology,37, 231–240. doi:10.1111/j.1600-0528.2009.00459.x 19302576 10.1111/j.1600-0528.2009.00459.x

[CIT0010] Hyde M. Wiggins R. D. Higgs P., & Blane D. B (2003). A measure of quality of life in early old age: The theory, development and properties of a needs satisfaction model (CASP-19).Aging & Mental Health,7, 186–194. doi:10.1080/1360786031000101157 12775399 10.1080/1360786031000101157

[CIT0011] Jivraj S. Nazroo J. Vanhoutte B., & Chandola T (2014). Aging and subjective well-being in later life.Journals of Gerontology. Series B: Psychological Sciences and Social Sciences,69, 930–941. doi:10.1093/geronb/gbu006 10.1093/geronb/gbu006 PMC4296137 24569002

[CIT0012] Katz S. Ford A. B. Moskowitz R. W. Jackson B. A., & Jaffe M. W (1963). Studies of illness in the aged. The index of ADL: A standardized measure of biological and psychological function.The Journal of the American Medical Association,185, 914–919. doi:10.1001/jama.1963.03060120024016 14044222 10.1001/jama.1963.03060120024016

[CIT0013] MacKinnon N. J., & Keating L. J (1989). The structure of emotions: Canada-United States comparisons.Social Psychology Quarterly,52, 70–83. doi.org/10.2307/2786905

[CIT0025] Marcenes, W., Kassebaum, N.J., Bernabé, E., Flaxman, A., Naghavi, M., Lopez, A., & Murray, C.J.L. (2013). Global Burden of Oral Conditions in 1990-2010: A Systematic Analysis.Journal of Dental Research,92, 592–597. doi: 10.1177/0022034513490168 23720570 10.1177/0022034513490168 PMC4484374

[CIT0014] O’Neil A. Berk M. Venugopal K. Kim S. W. Williams L. J., & Jacka F. N (2014). The association between poor dental health and depression: Findings from a large-scale, population-based study (the NHANES study).General Hospital Psychiatry,36, 266–270. doi:10.1016/j.genhosppsych.2014.01.009 24636212 10.1016/j.genhosppsych.2014.01.009

[CIT0015] Radloff L. S (1977). The CES-D scale: A self-report depression scale for research in the general population.Applied Psychological Measurement,1, 385–401. doi:10.1177/014662167700100306

[CIT0016] Rodrigues S. M. Oliveira A. C. Vargas A. M. Moreira A. N., & Ferreira E. F (2012). Implications of edentulism on quality of life among elderly.International Journal of Environmental Research and Public Health,9, 100–109. doi:10.3390/ijerph9010100 22470281 10.3390/ijerph9010100 PMC3315080

[CIT0017] Rouxel P. Tsakos G. Demakakos P. Zaninotto P. Chandola T., & Watt R. G (2015). Is social capital a determinant of oral health among older adults? Findings from the English longitudinal study of ageing.PLoS One,10, e0125557. doi:10.1371/journal.pone.0125557 25992569 10.1371/journal.pone.0125557 PMC4436243

[CIT0018] Steele J. G. Sanders A. E. Slade G. D. Allen P. F. Lahti S. Nuttall N., & Spencer A. J (2004). How do age and tooth loss affect oral health impacts and quality of life? A study comparing two national samples.Community Dentistry and Oral Epidemiology,32, 107–114. doi:10.1111/j.0301-5661.2004.00131.x 15061859 10.1111/j.0301-5661.2004.00131.x

[CIT0019] Steffick D. E (2000). Documentation of affective functioning measures in the Health and Retirement Study (HRS/AHEAD) Documentation Report DR-005 Retrieved from http://hrsonline.isr. umich.edu/sitedocs/userg/dr-005.pdf

[CIT0020] Takiguchi T. Yoshihara A. Takano N., & Miyazaki H (2015). Oral health and depression in older Japanese people.Gerodontology. doi:10.1111/ger.12177 10.1111/ger.12177 25645887

[CIT0021] Tsakos G. Marcenes W., & Sheiham A (2001). Evaluation of a modified version of the index of Oral Impacts On Daily Performances (OIDP) in elderly populations in two European countries.Gerodontology,18, 121–130. doi:10.1111/j.1741-2358.2001.00121.x 11794738 10.1111/j.1741-2358.2001.00121.x

[CIT0022] Vink D. Aartsen M. J., & Schoevers R. A (2008). Risk factors for anxiety and depression in the elderly: A review.Journal of Affective Disorders,106, 29–44. doi:10.1016/j.jad.2007.06.005 17707515 10.1016/j.jad.2007.06.005

[CIT0023] White D. A. Tsakos G. Pitts N. B. Fuller E. Douglas G. V. Murray J. J., & Steele J. G (2012). Adult Dental Health Survey 2009: Common oral health conditions and their impact on the population.British Dental Journal,213, 567–572. doi:10.1038/sj.bdj.2012.1088 23222333 10.1038/sj.bdj.2012.1088

