# Supplementary material for: Oral Health—A Neglected Aspect of Subjective Well-Being in Later Life
Source: J Gerontol B Psychol Sci Soc Sci. 2016 Mar 12;73(3):382–6. doi: 10.1093/geronb/gbw024 (PMC5927002; doi:10.1093/geronb/gbw024)
Supplement: Supplementary Tables 1-8 [file gbw024_suppl_supplementary_file.docx]

**Supplementary Data**

**Supplementary Table 1: Mean (sd) Change in Subjective Well-Being by Change in Oral Health and Covariates of ELSA Respondents between Waves 3 and 5**

*Notes*. ELSA = English Longitudinal Study of Ageing; CASP-19 = Quality of Life; OIDP = Oral Impacts on Daily Performances; ADL/IADL = Activities of Daily Living/Instrumental Activities in Daily Living.

**Supplementary Table 2: Multiple Regression Models of Change in CASP-19 Scores by Change in Oral Impacts between Waves 3 (2006-07) and 5 (2010-11), Regression Coefficient (95%CI)**

**Supplementary Table 3: Multiple Regression Models of Change in Life Satisfaction Scores by Change in Oral Impacts between Waves 3 (2006-07) and 5 (2010-11), Regression Coefficient (95%CI)**

**Supplementary Table 4: Multiple Regression Models of Change in Depressive Symptoms Scores by Change in Oral Impacts between Waves 3 (2006-07) and 5 (2010-11), Regression Coefficient (95%CI)**

**Supplementary Table 5: Multiple Regression Models of Change in CASP-19 Scores by Change in Edentulism between Waves 3 (2006-07) and 5 (2010-11), Regression Coefficient (95%CI)**

**Supplementary Table 6: Multiple Regression Models of Change in Life Satisfaction Scores by Change in Edentulism between Waves 3 (2006-07) and 5 (2010-11), Regression Coefficient (95%CI)**

**Supplementary Table 7: Multiple Regression Models of Change in Depressive Symptoms Scores by Change in Edentulism between Waves 3 (2006-07) and 5 (2010-11), Regression Coefficient (95%CI)**

**Supplementary Table 8: Multinomial Logit Models of Change in Depressive Symptom (CES-D cut-off 3) by Change in Oral Health Indicators between Waves 3 (2006-07) and 5 (2010-11), Relative Risk Ratios (RRRs) (95%CI)**

 *Notes*. CES-D = Center for Epidemiologic Studies-Depression; CASP-19 = Quality of Life; OIDP = Oral Impacts on Daily Performances.

The reference category for change in depressive symptoms is ‘no change in depressive symptoms’

Model 1: Bivariate model only including change in oral health

Model 2: Model 1 adjusted for socio-demographic (age-groups, gender, cohabiting status), socio-economic (economic activity status, wealth quintiles), health (self-rated health, ADL/IADL), smoking and psychosocial (social support) factors

***p<.*01; ***p<.001
